# Production of a New Cyclic Depsipeptide by the Culture Broth of *Staphylococcus* sp. Isolated from *Corallina* *officinalis* L.

**DOI:** 10.3390/metabo9110273

**Published:** 2019-11-11

**Authors:** Reda F. A. Abdelhameed, Sameh S. Elhady, Ahmad O. Noor, Diena M. Almasri, Alaa A. Bagalagel, Galal T. Maatooq, Amgad I. M. Khedr, Koji Yamada

**Affiliations:** 1 Department of Pharmacognosy, Faculty of Pharmacy, Suez Canal University, Ismailia 41522, Egypt; omarreda_70@yahoo.com; 2 Department of Natural Products and Alternative Medicine, Faculty of Pharmacy, King Abdulaziz University, Jeddah 21589, Saudi Arabia; ssahmed@kau.edu.sa; 3 Department of Pharmacognosy, Faculty of Pharmacy, Port Said University, Port Said 42526, Egypt; a_mansour7799@yahoo.com; 4 Department of Pharmacy Practice, Faculty of Pharmacy, King Abdulaziz University, Jeddah 21589, Saudi Arabia; aonoor@kau.edu.sa (A.O.N.); dalmasri@kau.edu.sa (D.M.A.); abagalagel@kau.edu.sa (A.A.B.); 5 Department of Pharmacognosy, Faculty of Pharmacy, The Islamic University in Najaf, Najaf 54001, Iraq; galaltm@yahoo.com; 6 Department of Pharmacognosy, Faculty of Pharmacy, Mansoura University, Mansoura 35516, Egypt; 7 Garden for Medicinal Plants, Graduate School of Biomedical Sciences, Nagasaki University; Bunkyo-machi 1-14, Nagasaki 852-8521, Japan

**Keywords:** *Corallina officinalis*, *Staphylococcus* sp., cyclic depsipeptide, antimicrobial assay

## Abstract

A new cyclic depsipeptide (**1**) has been isolated from culture broth of *Staphylococcus* sp. (No. P-100826-4-6) derived from *Corallina officinalis* L., together with the known compounds indol-3-carboxylic acid (**2**), 1,5-dideoxy-3-C-methyl arabinitol (**3**), thymine (**4**), uracil (**5**), cyclo (L-pro-L-omet) (**6**) and macrolactin B (**7**). The structure of (**1**) was established to be cyclo (2α, 3-diaminopropoinc acid-L-Asn-3-β-hydroxy-5-methyl-tetradecanoic acid-L-Leu^1^-L-Asp-L-Val-L-Leu^2^-L-Leu^3^) by extensive spectroscopic techniques including ^1^H NMR, ^13^C NMR, ^1^H‒^1^H COSY, HMBC, HSQC, NOESY, and HRFABMS. The antimicrobial activities of compounds **1–7** were evaluated. Compounds **1–5**, and **7** showed moderate antimicrobial activity while compound **6** exhibited a potent antimicrobial and antifungal activities.

## 1. Introduction

Bioprospecting studies of endophytic microorganisms play a principal part in the discovery of lead compounds for the improvement of drugs for the management of humanity’s diseases [1,2,3]. Marine microorganisms are widely recognized as promising sources of secondary metabolites [2,4,5,6]. These organisms, prosperous in diverse marine environments, have produced a wide variety of structurally exclusive and biologically active compounds that have attracted significant interest for biomedical researches [4,7,8,9].

In the last decade, a significantly increased interest in the isolation of bioactive secondary metabolites from marine microbes has been reported [10]. Peptides, which were derived from different organisms including marine microbes, represent an important chemical class with diverse structures and significant biological activities. The biological activity of marine-derived peptides has been shown to depend on the composition and sequence of amino acids, their structural properties, as well as on the environmental habitat for producer bacteria [11,12]. These substances are actively synthesized by marine microorganisms during their life cycle [13]. To date, lots of peptide metabolites, which are consisting of 20–40 amino acids, have been separated from various marine microorganisms [11,12,14]. Most of them are capable of quick inhibition or killing of a wide range of microbes. Other antimicrobial peptides (proteins consisting of 100 or more amino acids) disrupt the function or the structure of microbial cell membranes by binding to specific targets [15,16].

**Figure 1:**
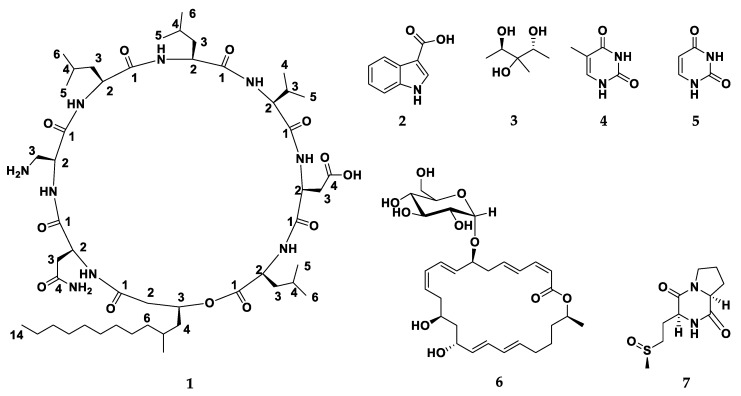
Structure of compounds **1**–**7**.

In our pursuit of isolation of natural compounds from marine sources [17,18,19,20], chemical investigation for the antimicrobial extract of *Staphylococcus* sp. No. [P-100826-4-6] derived, was carried out. This study led to discovery of a new natural cyclic depsipeptide (**1**) along with six known compounds; indol-3-carboxylic acid (**2**) [21], 1,5-dideoxy-3-C-methyl arabinitol (**3**) [22], thymine (**4**) [21], uracil (**5**) [23], cyclo (L-pro-L-omet) (**6**) [24], and macrolactin B (**7**) [25] (Figure 1). Therefore, structure identification of the isolated pure compounds **1**–**7** and their antimicrobial activity will be discussed.

## 2. Results and Discussion

### 2.1. Isolation Method of Compounds ***1**–**7***

The fermented broth of the marine bacterium *Staphylococcus* sp. No. [P-100826-4-6] was extracted with different organic solvents. Successive fractionation of the combined extracts was done using silica gel column chromatography based on increasing polarity, Sephadex LH-20, Diaion HP-20, reversed-phase C18 silica gel column; subsequently, final purification on a C18 RP-HPLC column gave seven compounds **1**–**7** (Figure 1).

### 2.2. Characterization of Compounds ***1**–**7*** Structures

**Figure 2:**
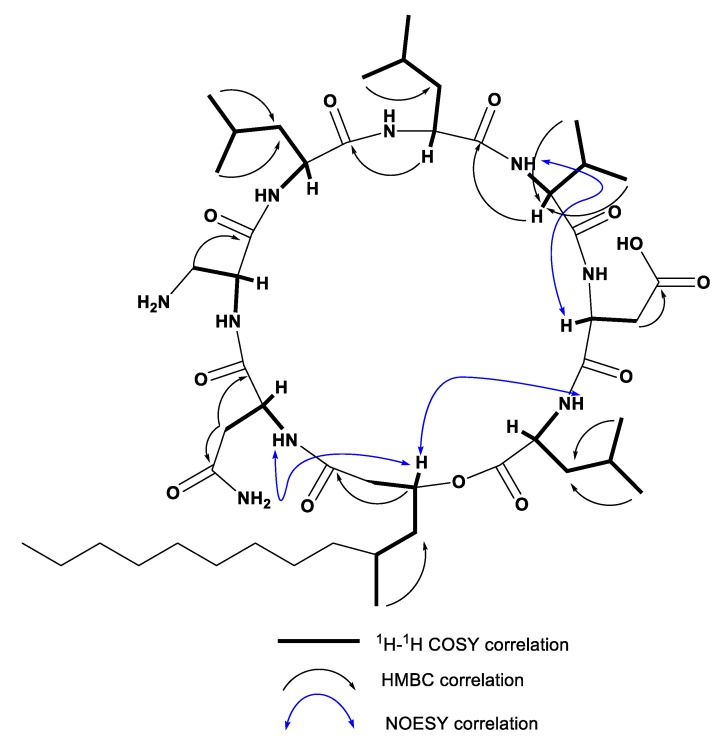
Selected correlations of COSY, HMBC, and NOESY observed for compound **1**.

Compound **1** (Figure 1) was obtained as a white amorphous powder by several chromatographic procedures obtained from the fermented broth of *Staphylococcus* species. It gave a [M + H]^+^ peak in the (FABMS) at *m/z* 994.7 and (HRFABMS) elemental composition [M + Na]^+^ at *m/z* 1016.6396 (calcd for C_49_H_87_N_9_NaO_12_, 1016.6372, *Δ*+2.5 mmu) (Figures S1–S3) indicating 11 degrees of unsaturation. The ^1^H NMR spectrum of **1** (Table 1, Figure S4) with HSQC and HMBC spectral data confirmed the existence of 8 methine protons (C-H) [*δ*_H_ 5.64, 5.64, 5.05, 4.98, 4.90, 4.83, 4.70, 4.62, 1H each], 7 amide protons NH-C=O [*δ*_H_ 9.58, 9.48, 9.20, 8.91, 8.83, 8.75, 8.41, partially overlapped, 1H each], 10 methyl groups, among them 9 methyl doublets [*δ*_H_ 1.17, 1.13, 1.02, 0.96, 0.95, 0.93, 0.92, 0.85, 3H each, d, *J* = 6.6 Hz], one methyl triplet [*δ*_H_ 0.81, 3H, t, *J* = 6.9 Hz] and long methylene chain centered at *δ*_H_ 1.22 (12H, brs). The ^13^C NMR and DEPT spectra of **1** (Table 1, Figure S5) with HSQC and HMBC spectral data exhibited 49 signals, attributable to 10 carbonyl carbons [*δ*_C_ 175.6, 175.5, 174.8, 173,8, 173.6, 173.4, 172.5, 172.4, 172.0, and 171.7], 7 bearing nitrogen methines [*δ*_C_ 61.0, 55.0, 53.5, 52.7, 52.5, 52.5, and 51.5], one oxymethine [*δ*_C_ 72.5], 5 methines [*δ*_C_ 28.5, 25.4, 25.3, 25.1, and 24.9] 10 methyls [*δ*_C_ 23.6, 23.4, 23.2, 22.8, 21.8, 21.5, 21.5, 19.5, 18.7, and 14.4], 16 methylenes (Table 1). We could easily deduce that **1** was depsipeptide composed of 1 Val, 3 Leu, 1 Asn, 1 Asp, and 1 A_2_pr residues interlinked with 3-hydroxy-5-methyl fatty acid by analysis of HSQC, HMBC, COSY and NOESY (Figures S6–S9) correlations as shown in Figure 2. The sequence of amino acids in **1** was identified by NOESY and HMBC data analysis for **1** as in Figure 2. NOESY correlations found in **1** between NH protons and adjacent amino acids methine protons clearly identified the following amide bonds: Asp-CO/Val-NH (*δ*_H_ 4.83/9.48), HMTDA-CO/Asn-NH (*δ*_H_ 5.64/8.91), HMTDA-CO/Leu^1^-NH (*δ*_H_ 5.64/8.41). The connectivity between HMFA and Leu^1^ was also identified by NOESY correlation between α-methine proton HMFA and NH proton of Leu^1^ (*δ*_H_ 5.64/8.41) and Asn (*δ*_H_ 5.64/8.91). These data, together with HMBC correlations as shown in Figure 2, finally enable us to set up the structure of **1** as cyclo (-A_2_Pr-Asn-HMTDA-Leu^1^-Asp-Val-Leu^2^-Leu^3^-). FABMS data of **1** (Figures S2 and S3) supported the amino acid sequence of **1** as shown in Table 2. The amino acids absolute configuration of **1** was recognized by the reaction of Marfey’s reagent [26,27] with the crude hydrolysate followed by co-injection of standard amino acids using HPLC analysis. The hydrolysate was recognized to possess 3 L-Leu, 1 L-Val, and 1 L-Asp. The configuration of A_2_Pr and C-3 of HMTDA were proposed by cautions analysis of NOESY correlations, as shown in Figure 2. The NOESY correlation between β-proton (*δ*_H_ 5.64) of HMTDA and amide proton (*δ*_H_ 8.91) of Asn, between amide proton (*δ*_H_ 8.91) of Asn and α-proton (*δ*_H_ 4.90) of Asn, between amide proton (*δ*_H_ 9.20) of A_2_Pr and α-proton (*δ*_H_ 5.64) of A_2_Pr, between amide proton (*δ*_H_ 9.58) of Leu^3^ and α-proton (*δ*_H_ 4.62) of Leu^3^, between amide proton (*δ*_H_ 8.75) of Leu^2^ and *α*-proton (*δ*_H_ 4.98) of Leu^2^, between amide proton (*δ*_H_ 9.48) of Val and *α*-proton (*δ*_H_ 4.70) of Val, revealed that the configuration at A_2_Pr and C-3 of HMTDA have the configuration of L-Leu, L-Val, and L-Asp. Therefore, the final structure of **1** was lastly characterized as cyclo (2*α*, 3-diamino-propoincacid-L-Asn-3-β-hydroxy-5-methyl-tetradecanoicacid-L-Leu^1^-L-Asp-L-Val-L-Leu^2^-L-Leu^3^).

The other known compounds **2**–**7**, were established by analysis of their spectroscopic data (MS and NMR), and with comparison of these data with those mentioned in the literature to be: indol-3-carboxylic acid (**2**) [21], 1,5-dideoxy-3-C-methyl arabinitol (**3**) [22], thymine (**4**) [21], uracil (**5**) [23], cyclo (L-pro-L-omet) (**6**) [24], and macrolactin B (**7**) [25] as shown in Figure 1.

### 2.3. Biological Activities of the Pure Compounds ***1**–**7***

The antimicrobial activities (Table 3) of compounds **1**–**7** were examined for their growth inhibition of 6 microorganisms including Gram-negative, Gram-positive bacteria and fungi using paper disk method with replication (*n* = 2). The antimicrobial activities were studied in a concentration of 100 μg/disk. As a result, compounds **1**–**5**, and **7** showed moderate activity against *Schizophyllum commune, Staphylococcus aureus* subsp. *aureus,* and *Escherichia coli,* with inhibition zones between 9 and 13, while compound **6** exhibited a significant antifungal activity against *Aspergillus niger*, *Penicillum crustosum*, and *Schizophyllum commune*, with inhibition zones of 16, 18 and 23. Furthermore, compound **6** showed antibacterial activity against *Staphylococcus aureus* subsp. *aureus*, *Pseudomonas aeruginosa* and *Escherichia coli*, with inhibition zones of 20, 25 and 21 as shown in Table 3.

The minimum inhibitory concentration (MIC) of compound **6** were evaluated. As a result, compound **6** exhibited a potent antifungal activity against *Aspergillus niger*, *Penicillum crustosum*, and *Schizophyllum commune* with MIC value of 50 μg/mL. Additionally, compound **6** showed antibacterial activity against *Staphylococcus aureus* subsp. *aureus*, *Pseudomonas aeruginosa* and *Escherichia coli*, with MIC value of 100 μg/mL.

## 3. Materials and Methods

### 3.1. General Experimental Procedures

Optical rotations were considered using JASCO DIP-370 digital polarimeter. IR spectra were obtained with JASCO FT/IR-410 spectrophotometers. ^1^H and ^13^C NMR, HSQC, ^1^H-^1^H COSY, HMBC, and NOESY spectra were obtained using Unity plus 500 spectrometer (Varian Inc., Palo Alto, CA, USA) operating at 125 MHz for ^13^C and 500 MHz for ^1^H. Chemical shifts of ^1^H-NMR and ^13^C NMR are expressed in *δ* values referring to the solvent peak *δ*_H_ 7.19, 7.55 and 8.71, *δ*_C_ 123.5, 135.5 and 149.9 for pyridine-*d*_5_, and coupling constants are expressed in Hz. TLC was carried out on aluminum-backed plates (Merck, Kieselgel 60 F_254_, 0.25 mm) and RP-18 F_254s_ plates (Merck). Si-gel F254 with a particle size of 0.0045–0.075 mm mesh (Wako Pure Chemical Industries Ltd., Osaka, Japan), Cosmosil 5C18-140 PREP (Nacalai tesque, No.379-34), and Sephadex LH-20 (Sigma-Aldrich, Darmstadt, Germany) were used as stationary phases for column chromatography. High resolution FABMS were obtained using JMS DX-303 spectrometer (JEOL Ltd., Tokyo, Japan). Preparative HPLC was utilized using a Develosil C-30-UG-5 (250 × 4.6 mm i.d Nomura Chemical Co., Aichi, Japan) adjusting the rate of flow at 1.5 mL/min, and a TOSOH RI-8020 detector.

### 3.2. Biological Materials

**Figure 3:**
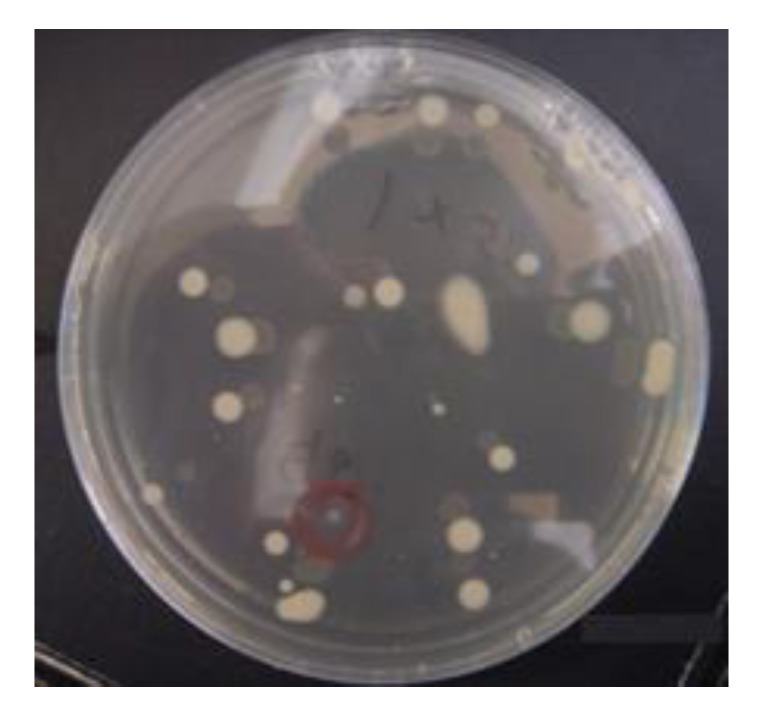
*Staphylococcus* sp. (No. P-100826-4-6) derived from *Corallina officinalis* L.

The isolated strain *Staphylococcus* sp. P-100826-4-6 (Figure 3) was obtained from *Corallina officinalis,* collected in Nagasaki Shitsu coast, Japan, in 2010. The voucher specimen was maintained at Garden for Medicinal Plants, Graduated School of Biomedical Sciences, Nagasaki University. *Staphylococcus* sp. was cultured on a medium based on sea water composed of (0.1% MgSO_4_, 0.3% KH_2_PO_4_, 0.3% yeast extract, 0.5% polypeptone, 1% glucose, 25% distilled water, 75% sea water) for 28 days at 25 °C on a rotary shaker. After culturing, sonication and filtration of the broth (32L) was conducted.

### 3.3. Purification of Compounds ***1**–**7***

After filtration, the broth was extracted three times with 10 L EtOAc. The EtOAc extract was completely dried to produce EtOAc extract (5.2 g). The water layer was subjected to Diaion HP-20 column eluting with water, 60% MeOH, 100% MeOH, and acetone correspondingly to yield eluted fractions of 60% MeOH (28.3 g) and 100% MeOH (8.0 g), and acetone (3.9 g). The EtOAc extract was chromatographed on silica gel column using CHCl_3_:MeOH; 10:0~0:10 to yield 25 fractions.

The 15th fraction (96 mg) was subjected to Sephadex LH-20 using CHCl_3_:MeOH; 1:1 as eluent to yield four sub-fractions (fractions A–D). Fraction D (10 mg) was purified on ODS column using 50% MeOH:H_2_O to give compound **2** (7 mg).

The 18th fraction (198 mg) was subjected to Sephadex LH-20 CHCl_3_:MeOH; 1:1 to give three fractions (fractions A–C). Fraction B (125 mg) was chromatographed on ODS column using 50% MeOH:H_2_O followed by final purification on Sephadex LH-20 eluted with CHCl_3_:MeOH; 1:1 to obtain compound **3** (86 mg). Fraction C (15 mg) was subjected to RP-HPLC Develosil C-30 using 55% MeOH:H_2_O to give compound **4** (8 mg).

The 20th fraction (476 mg) was subjected to Sephadex LH-20 eluted with CHCl_3_:MeOH; 1:1 to give three sub-fractions (fractions A–C). Fraction A (290 mg) was chromatographed on silica gel column with CHCl_3_:MeOH; 10:0 ~ 0:10 as eluent to afford three fractions. Fraction A-1 (50 mg) was finally purified on RP-HPLC Wakosil 5C-18 using 70% MeOH-H_2_O to afford compound **1** (8 mg). Fraction C (35mg) was recrystallized from MeOH to yield compound **5** (31 mg). Dissolution of the 100% MeOH fraction (8.0 g) was done using CHCl_3_:MeOH:H_2_O; 5:5:1 to afford soluble fraction (4.7 g). The soluble fraction was subjected to Sephadex LH-20 using CHCl_3_:MeOH; 1:1 to obtain seven sub-fractions (fractions A–G). Fraction D (587 mg) was chromatographed on ODS column using MeOH:H_2_O gradient elution to give seven fractions. Fraction D-1 (45 mg) was chromatographed on Sephadex LH-20 using CHCl_3_:MeOH; 1:1 isochratic elution to obtain two fractions (fractions D-1a and D-3b). Medium pressure liquid chromatography (MPLC) used for final purification of fraction D-3b (17 mg) using CHCl_3_:MeOH; 98:2 as eluent to give compound **6** (5.5 mg). Fraction D-6 (95 mg) was subjected to silica gel column using gradient elution of CHCl_3_:MeOH to give four fractions (fractions D-6a–D-6d). Fraction D-6c (40 mg) was finally isolated on Sephadex LH-20 using CHCl_3_:MeOH; 1:1 to obtain compound **7** (26.7 mg).

**Compound 1**: Cyclo (2*α*, 3-diamino-propoinc acid-L-Asn-3-β-hydroxy-5-methyl-tetradecanoic acid-L-Leu^1^-L-Asp-L-Val-L-Leu^2^-L-Leu^3^): White amorphous powder ${\left[\alpha \right]}_{D}^{30}$**‒** 29.8^º^ (*c* = 0.01, pyridine); IR *ν*_max_ (dry film) 3318, 2965, 2862, 1733, 1684 cm^−1^; ^1^H and ^13^C NMR data (see Table 1); (HRFABMS) elemental composition [M + Na]^+^ at *m/z* 1016.6396 (calcd for C_49_H_87_N_9_NaO_12_, 1016.6372, Δ+2.5 mmu), (+) FABMS *m*/*z*: 994.7 [M + H]^+^ (Figures S1–S3).

### 3.4. Configuration of Amino Acids 

Hydrolysis of 1.5 mg of compound **1** was achieved using 1 mL of 6 N HCl for 16 h at 110 °C. Concentration for the resulting hydrolysate was followed by complete dryness under a vacuum to afford a residue. Dissolution of the solid residue was achieved in 50 μL of pure H_2_O and 40 μL of 1 M NaHCO_3_ aq. and 100 μL of 1% of (2S)-2-(5-fluoro-2,4-dinitroanilino)-4-methylpentanamide (FDLA) dissolved in acetone. Heating of the formed mixture was performed for 1 h at 37 °C, followed by adding 20 μL of 1 N HCl. A yellow solid was formed after complete drying of the previously prepared solution. The resulting solid was dissolved in 40% MeCN:H_2_O (500 μL) and co-injected with standard d- and l- amino acids using RP-HPLC (UV detector: 340 nm, flow rate: 1 mL/min, mobile phase: 40% MeCN:H_2_O).

### 3.5. Antimicrobial Activity of Compounds ***1**–**7***

Antimicrobial activities of the pure compounds were checked using paper disk methods [28,29] against *Staphylococcus aureus* subsp. *aureus*, *Escherichia coli*, *Pseudomonas aeruginosa*, *Aspergillus niger*, *Schizophyllum commune*, and *Penicillium crustosum*, with concentration 100 and 50 μg/disk. Determination of the MIC for compound **6** was achieved by using tube-dilution method [30].

## 4. Conclusions

Chemical study of the antimicrobial extracts obtained from *Staphylococcus* sp. derived from *Corallina officinalis* L., yielded a new cyclic depsipeptide (**1)** along with the known compounds indol-3-carboxylic acid (**2**), 1,5-dideoxy-3-C-methyl arabinitol (**3**), thymine (**4**), uracil (**5**), cyclo (L-pro-L-omet) (**6**), and macrolactin B (**7**). The structure of the isolated compounds were elucidated by extensive spectroscopic methods including (^1^H NMR, ^13^C NMR, COSY, HMBC, HSQC, NOESY, HRFABMS, and IR). The antimicrobial activities of the isolated compounds were evaluated. Compounds **1**–**5**, and **7** slightly showed an inhibition zone against *Schizophyllum commune, Escherichia coli,* and *Staphylococcus aureus* subsp. *aureus,* while compound **6** exhibited a potent antifungal activity against *Aspergillus niger*, *Penicillum crustosum*, and *Schizophyllum commune*, with MIC value 50 μg/mL. Additionally, compound **6** showed antibacterial activity against *Escherichia coli*, *Staphylococcus aureus* subsp. *aureus*, and *Pseudomonas aeruginosa* with MIC 100 μg/mL.

## Figures and Tables

**Table 1:** ^1^H and ^13^C NMR data of compound **1** (pyridine-*d*_5_) ^a^.

Position	*δ*_H_ (m, *J* in Hz)	*δ*_C_	Position	*δ*_H_ (m, *J* in Hz)	*δ*_C_
L-**Leu^1^**			2	4.62 m	52.5 (CH)
1	-	173.4 (C)	3	2.10 m	40.0 (CH_2_)
2	5.05 m	52.7 (CH)	4	1.97 m	25.1 (CH)
3	1.97, 2.10 m	39.5 (CH_2_)	5	1.02 d (6.6)	23.4 (CH_3_)
4	1.97 m	24.9 (CH)	6	0.95 d (6.6)	21.5 (CH_3_)
5	0.91 d (6.6)	21.3 (CH_3_)	NH	9.58 br s	-
6	0.85 d (6.6)	22.8 (CH_3_)	**A_2_Pr**		
NH	8.41 br s		1	-	173.8 (C)
L-**Asp**			2	5.64 m	51.5 (CH)
1	-	172 (C)	3	3.62 dd (15.8, 8.8) 3.40 dd (15.8, 4.8)	37.3 (CH_2_)
2	4.83 m	53.5 (CH)	NH	9.20 br s	-
3	1.97, 2.67 m	33.7 (CH_2_)	NH_2_	Not observed	-
4		175.6 (C)	L-**Asn**		
NH	8.83 (1H, brs)	-	1	-	173.6 (C)
L-**Val**			2	4.90 m	55.0 (CH)
1	-	172.5 (C)	3	2.67,2.90 m	35.0 (CH_2_)
2	4.70 t (6.6)	61.0 (CH)	4	-	175.5 (C)
3	2.67 m	28.5 (CH)	NH	8.91 br s	-
4	1.17 d (6.6)	19.5 (CH_3_)	NH_2_	Not observed	-
5	1.13 d (6.6)	18.7 (CH_3_)	**HMTDA**		
NH	9.48 br s	-	1	-	171.7 (C)
L-**Leu^2^**			2	2.94, 2.90 m	43.0 (CH_2_)
1	-	172.4 (C)	3	5.64 m	72.5 (CH)
2	4.98 q (7.9)	52.5 (CH)	4	1.76 m	42.6 (CH_2_)
3	2.10 m	39.6 (CH_2_)	5	1.40 m	25.4 (CH)
4	1.97 m	25.3 (CH)	5Me	0.93 d (6.6)	23.6 (CH_3_)
5	0.96 d (6.6)	23.2 (CH_3_)	6	1.29 (overlapped)	32.0 (CH_2_)
6	0.92 d (6.6)	21.8 (CH_3_)	7–12	1.22 m	28.7–29.0 (CH_2_)
NH	8.75 br s	-	13	1.33 (overlapped)	22.9 (CH_2_)
L-**Leu^3^**			14	0.81 t (6.9)	14.4 (CH_3_)
1	-	174.8 (C)			

^a^ Spectra were acquired at 23 °C. Chemical shifts were given in *δ* (ppm).

**Table 2:**
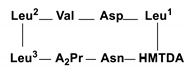
(+) FABMS parent and fragment ions of compound **1**.

Fragments	*m*/*z*
[Leu^3^–A_2_Pr–Asn–HMTDA–Leu^1^–Asp–Val–Leu^2^]^+^	994.7
[Leu^3^–A_2_Pr–Asn–HMTDA–Leu^1^–Asp–Val]^+^	881.6
[A_2_Pr–Asn–HMTDA–Leu^1^–Asp]^+^	669.6
[A_2_Pr–Asn–HMTDA–Leu^1^]^+^	554.6
[A_2_Pr–Asn–HMTDA]^+^	441.4
[A_2_Pr–Asn]^+^	201.2

**Table 3:** Antimicrobial activities of compounds **1**–**7**.

Compound	Inhibition Zone (mm, 100 µg/disc)					
	*S. aureus*	*E. coli*	*P. aeruginosa*	*S. commune*	*P. crustosum*	*A. niger*
**1**	13	11	NA	11	NA	NA
**2**	11	9	NA	13	NA	NA
**3**	10	13	NA	12	NA	NA
**4**	10	12	NA	11	NA	NA
**5**	12	11	NA	10	NA	NA
**6**	20	21	25	23	18	16
**7**	13	10	NA	13	NA	NA
**Cipro** **fl** **oxacin ^a^**	23	24	29			
**Nystatin ^b^**				23	20	19

^a^ positive antibacterial control (50 µg/disc); ^b^ positive antifungal control (100 µg/disc).

## Supplementary Materials

The following are available online at https://www.mdpi.com/2218-1989/9/11/273/s1, Figure S1: Elemental composition of compound 1, Figure S2: FABMS spectrum of compound 1, Figure S3: FABMS fragments of compound 1, Figure S4: ^1^H-NMR spectrum of compound 1 (pyridine-d_5_), Figure S5: ^13^C-NMR spectrum of compound 1 (pyridine-d_5_), Figure S6: HSQC spectrum of compound 1 (pyridine-d_5_), Figure S7: HMBC spectrum of compound 1 (pyridine-d_5_), Figure S8: ^1^H-^1^H COSY spectrum of compound 1 (pyridine-d_5_), Figure S9: NOESY spectrum of compound 1 (pyridine-d_5_).
