# Supplementary material for: Production of a New Cyclic Depsipeptide by the Culture Broth of Staphylococcus sp. Isolated from Corallina officinalis L
Source: Metabolites. 2019 Nov 11;9(11):273. doi: 10.3390/metabo9110273 (PMC6918221; doi:10.3390/metabo9110273)
Supplement: Supplementary file 1 [file metabolites-09-00273-s001.pdf]

# Production of A New Cyclic Depsipeptide by the Culture Broth of *Staphylococcus* sp. Isolated from *Corallina officinalis* L.

**Reda F. A. Abdelhameed [1], Sameh S. Elhady [2,3], Ahmad O. Noor [4], Diena M. Almasri [4], Alaa A. Bagalagel [4], Galal T. Maatooq [5,6], Amgad I. M. Khedr [3] and Koji Yamada [7,*]**

**Additional Experimental Detail.**

1) **Figure S1**. Elemental composition of compound **1**
2) **Figure S2**. FABMS spectrum of compound **1**
3) **Figure S3**. FABMS fragments of compound **1**
4) **Figure S4**. $^1$H-NMR spectrum of compound **1** (pyridine-$d_5$)
5) **Figure S4-a**. $^1$H-NMR spectrum of compound **1** with identified signals
6) **Figure S5**. $^{13}$C-NMR spectrum of compound **1** (pyridine-$d_5$)
7) **Figure S6**. HSQC spectrum of compound **1** (pyridine-$d_5$)
8) **Figure S7**. HMBC spectrum of compound **1** (pyridine-$d_5$)
9) **Figure S7a-S7-b**. HMBC spectrum expansions of compound **1** (pyridine-$d_5$)
10) **Figure S8**. $^1$H-$^1$H COSY correlations of compound **1** (pyridine-$d_5$)
11) **Figure S8-a**. $^1$H-$^1$H COSY spectrum of compound **1** with identified correlations
12) **Figure S9**. NOESY correlations of compound **1** (pyridine-$d_5$)
13) **Figure S9-a**. NOESY spectrum of compound **1** with identified correlations

```
[ Elemental Composition ]                                        Page: 1
Data   : DHFA2493                         Date : 06-Mar-2013 14:10
Sample: Mansour       No.A-20-1-2-2
Note : No.2             Pyridine   +      M.B    +    NaCl
Inlet : Direct                            Ion Mode : FAB+
RT : 0.67 min                             Scan#: (6,7)
Elements : C 49/0, H 90/0, N 9/0, O 12/0, Na 1/0
Mass Tolerance      : 10mmu
Unsaturation (U.S.) : -5.0 - 60.0

Observed m/z  Int%    Err[ppm / mmu]    U.S.  Composition
 1017.6417     9.1      -3.2 /  -3.3    10.0  C 49 H 88 N 9 O 12 Na

 1016.6396    14.1      +2.4 /  +2.5    10.5  C 49 H 87 N 9 O 12 Na
```

**Figure S1**. Elemental composition of compound **1**

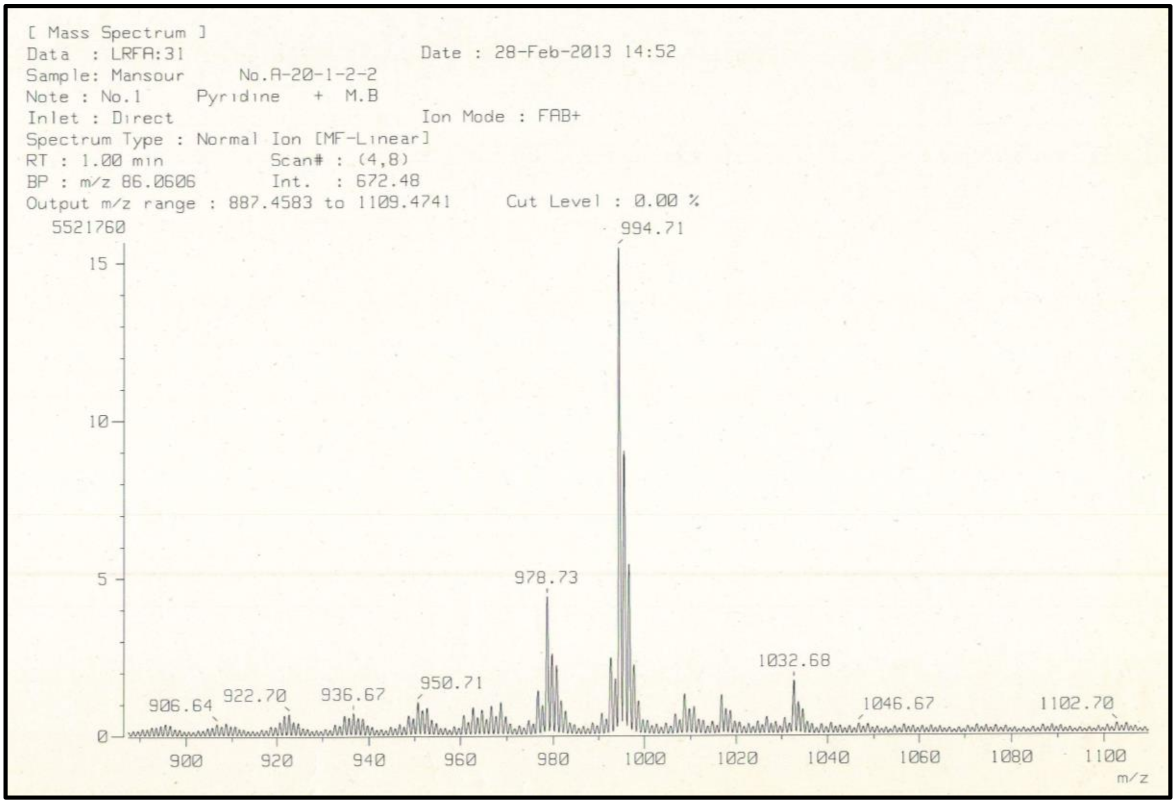


**Figure S2**. FABMS spectrum of compound **1**

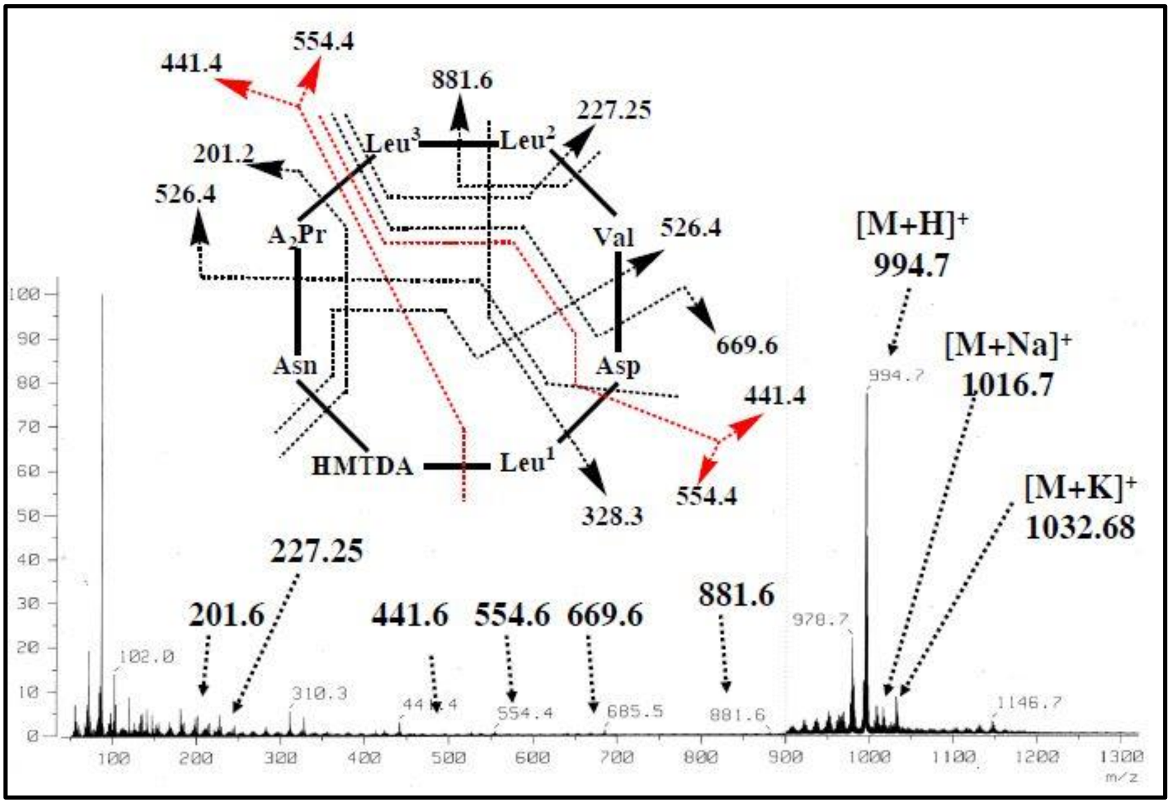


**Figure S3**. FABMS fragments of compound **1**

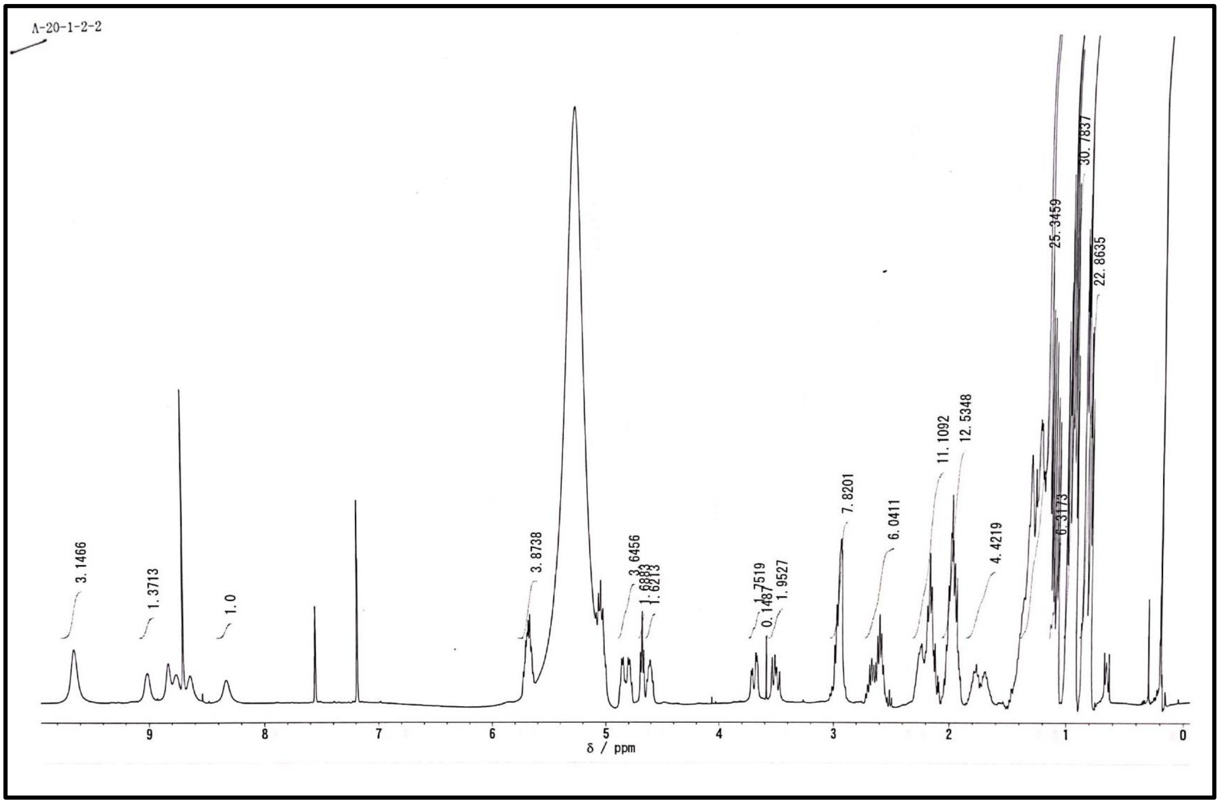


**Figure S4**. $^{1}$H-NMR spectrum of compound **1** (pyridine-$d_5$)

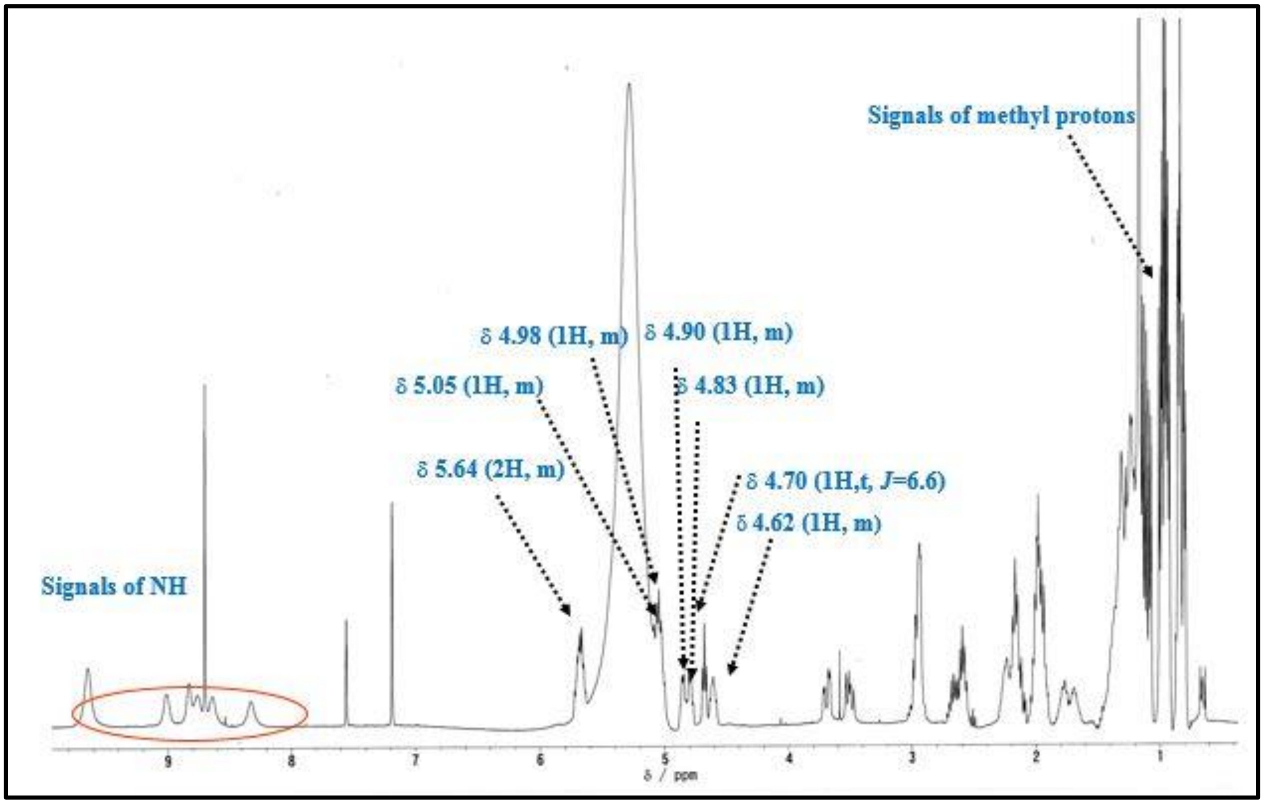


**Figure S4-a**. $^{1}$H-NMR spectrum of compound **1** with identified signals

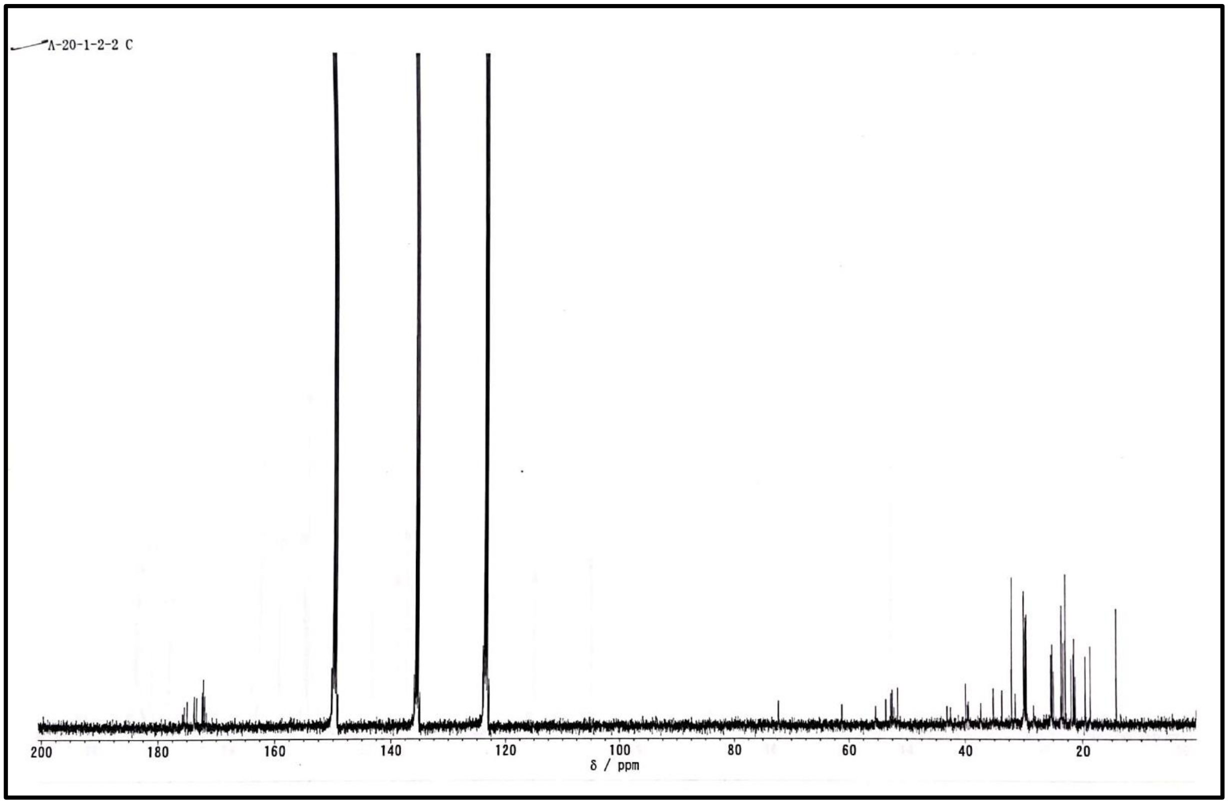


**Figure S5**. $^{13}$C-NMR spectrum of compound **1** (pyridine-$d_5$)

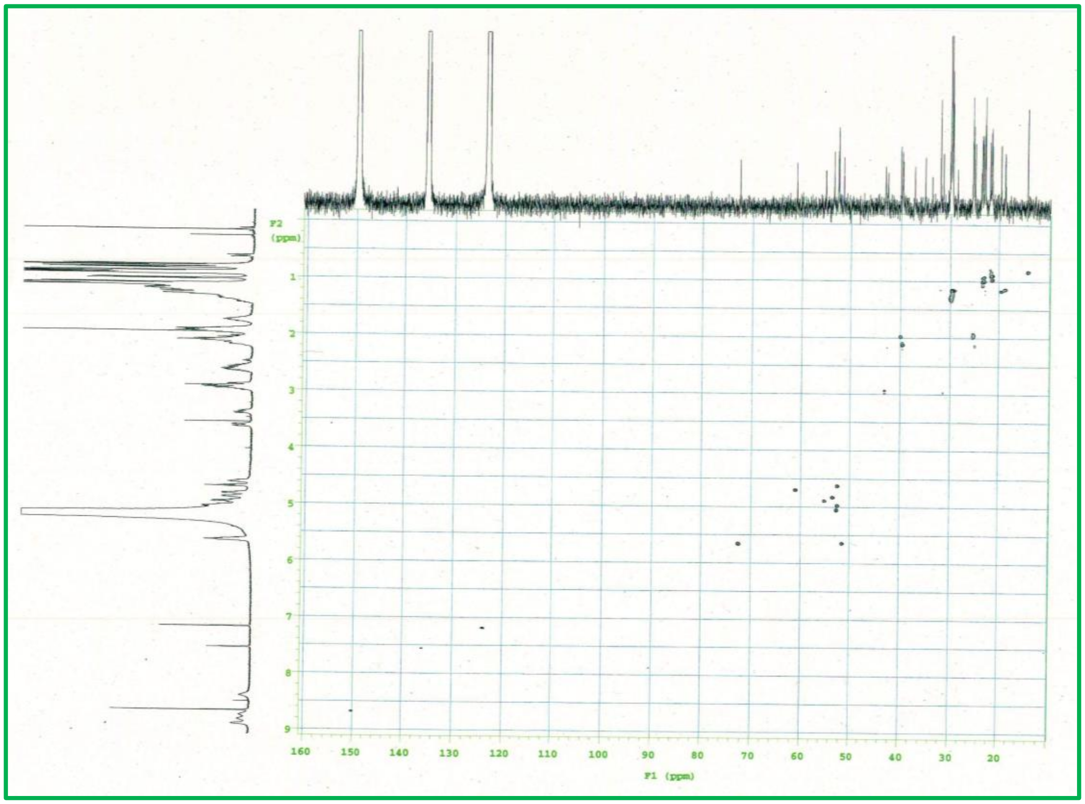


**Figure S6**. HSQC spectrum of compound **1** (pyridine-$d_5$)

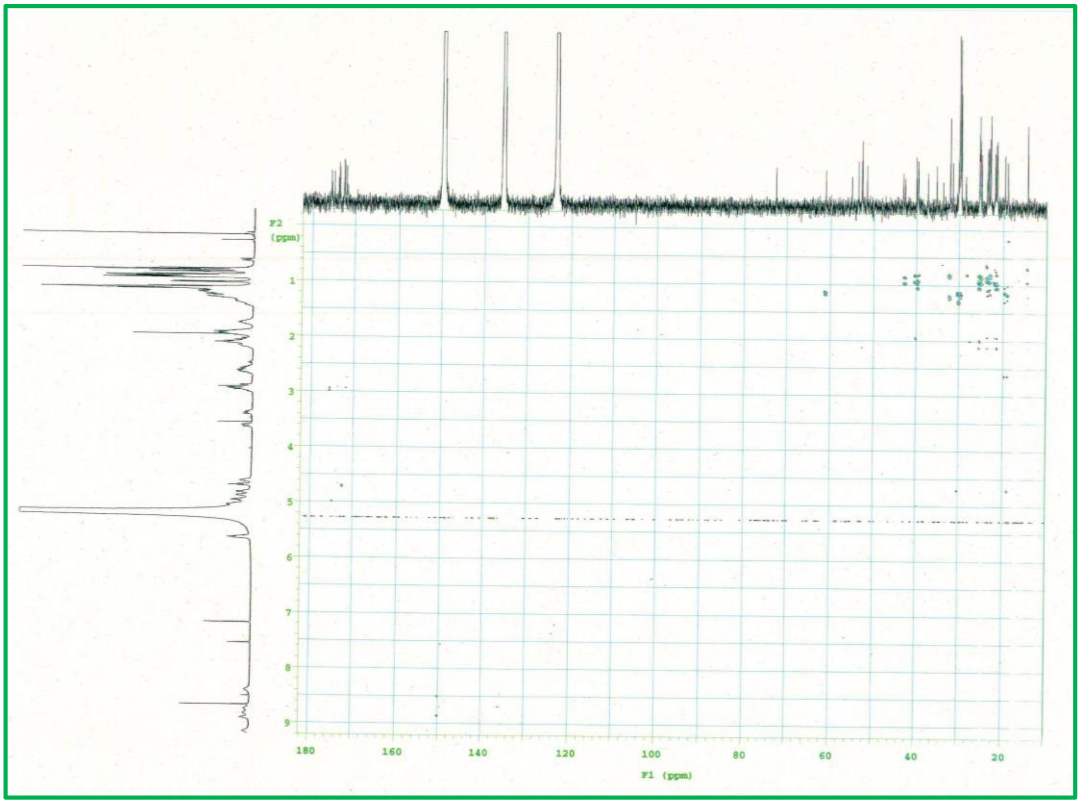



**Figure S7**. HMBC spectrum of compound **1** (pyridine-$d_5$)

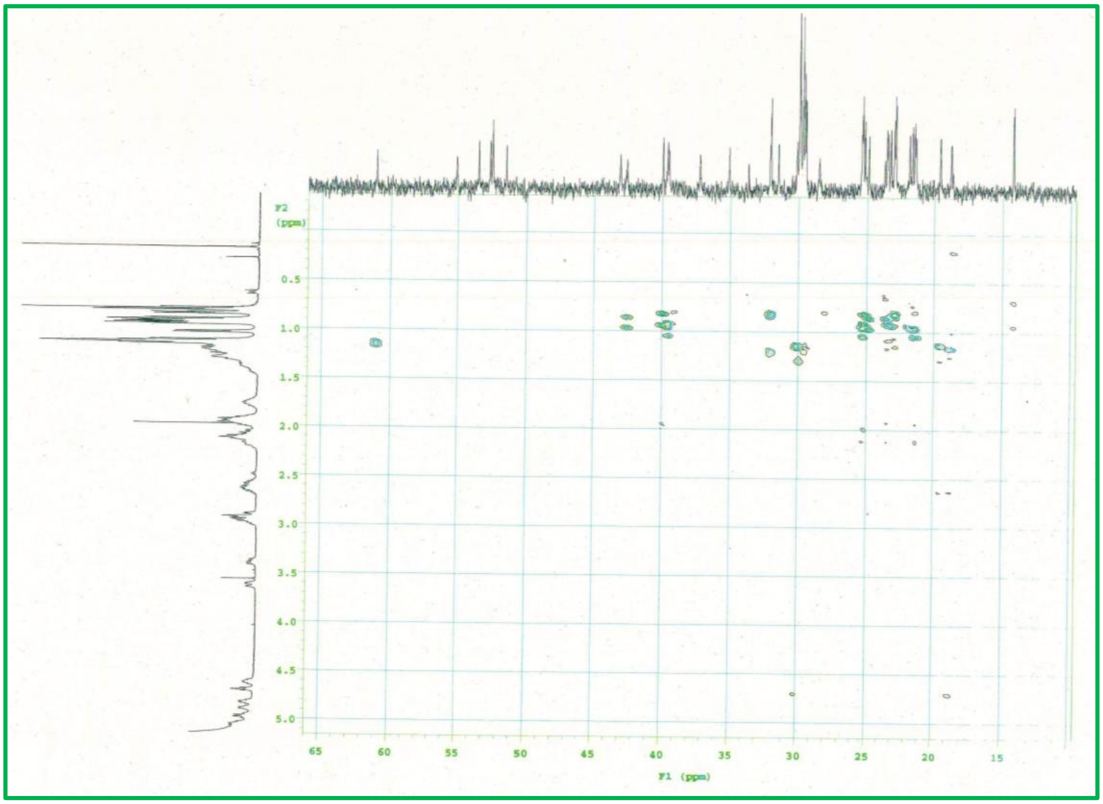



**Figure S7-a**. HMBC spectrum expansions of compound **1** (pyridine-$d_5$)

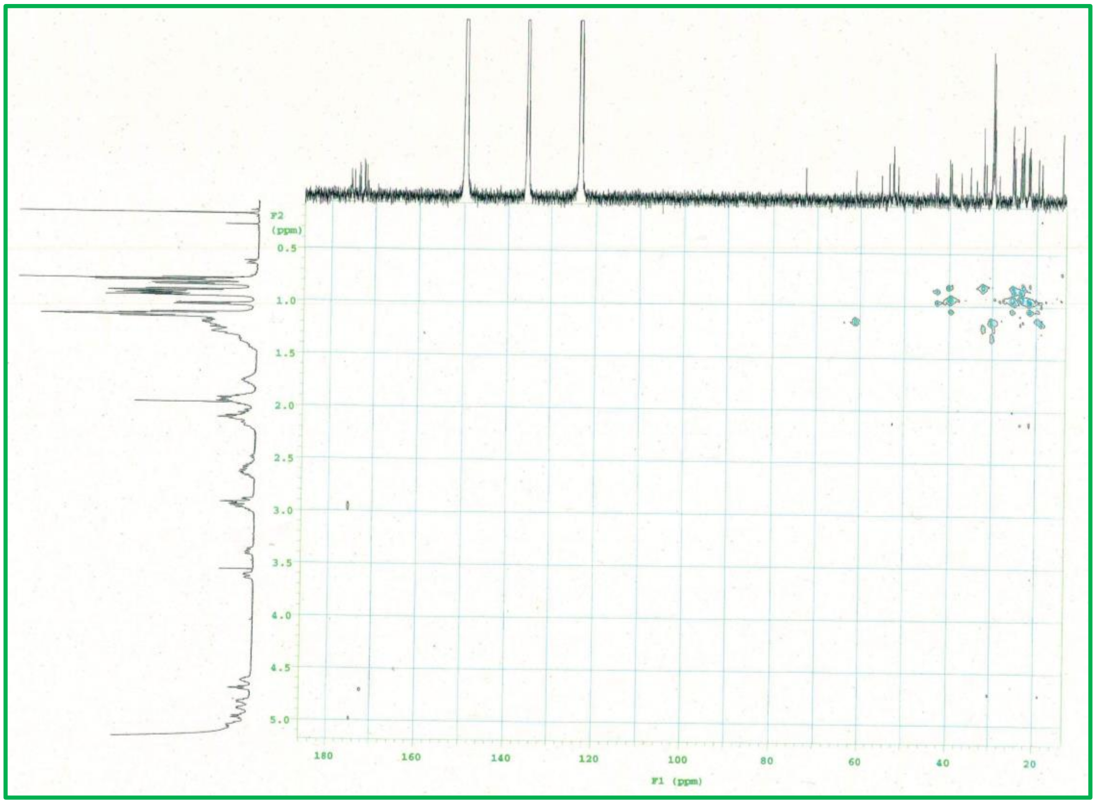


**Figure S7-b**. HMBC spectrum expansions of compound **1** (pyridine-$d_5$)

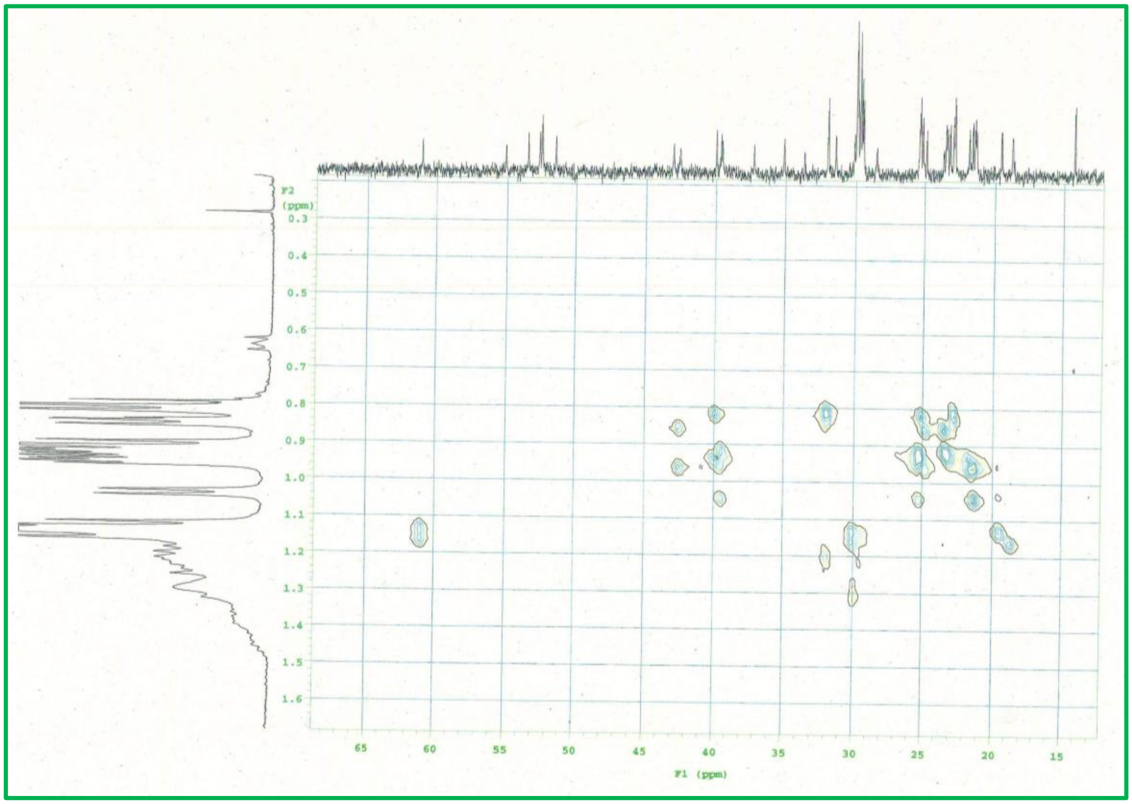


**Figure S7-c**. HMBC spectrum expansions of compound **1** (pyridine-$d_5$)

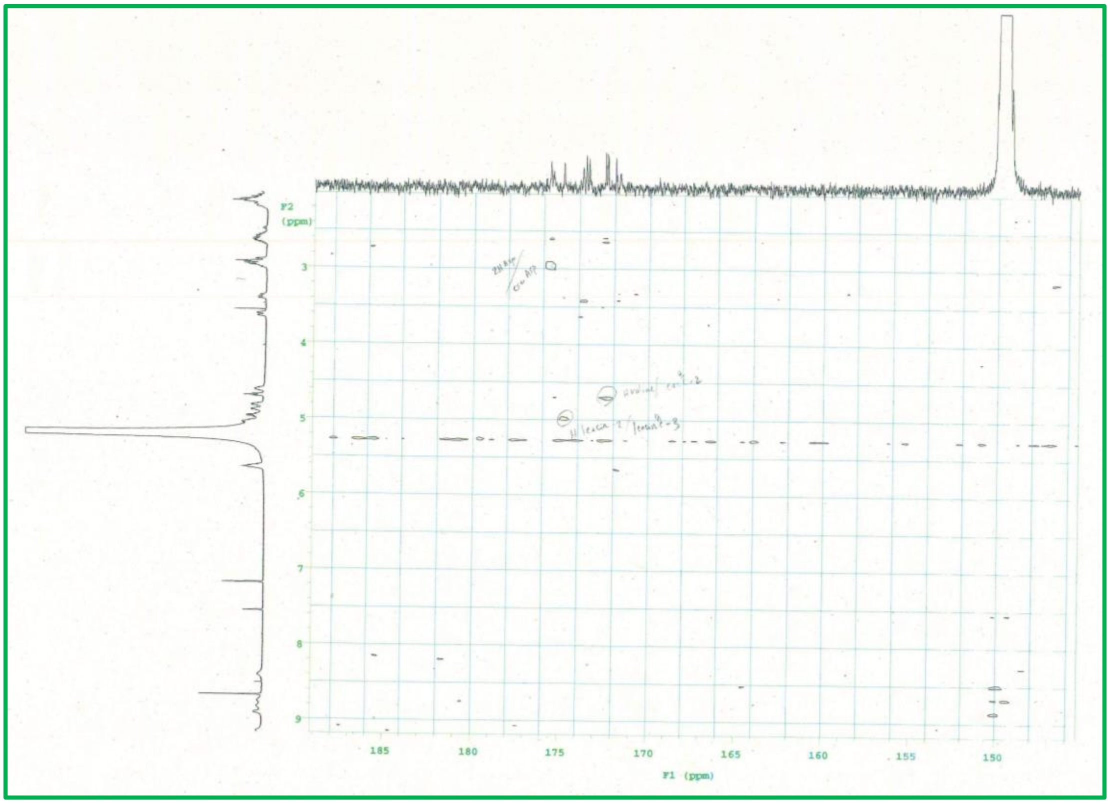


**Figure S7-d**. HMBC spectrum expansions of compound **1** (pyridine-$d_5$)

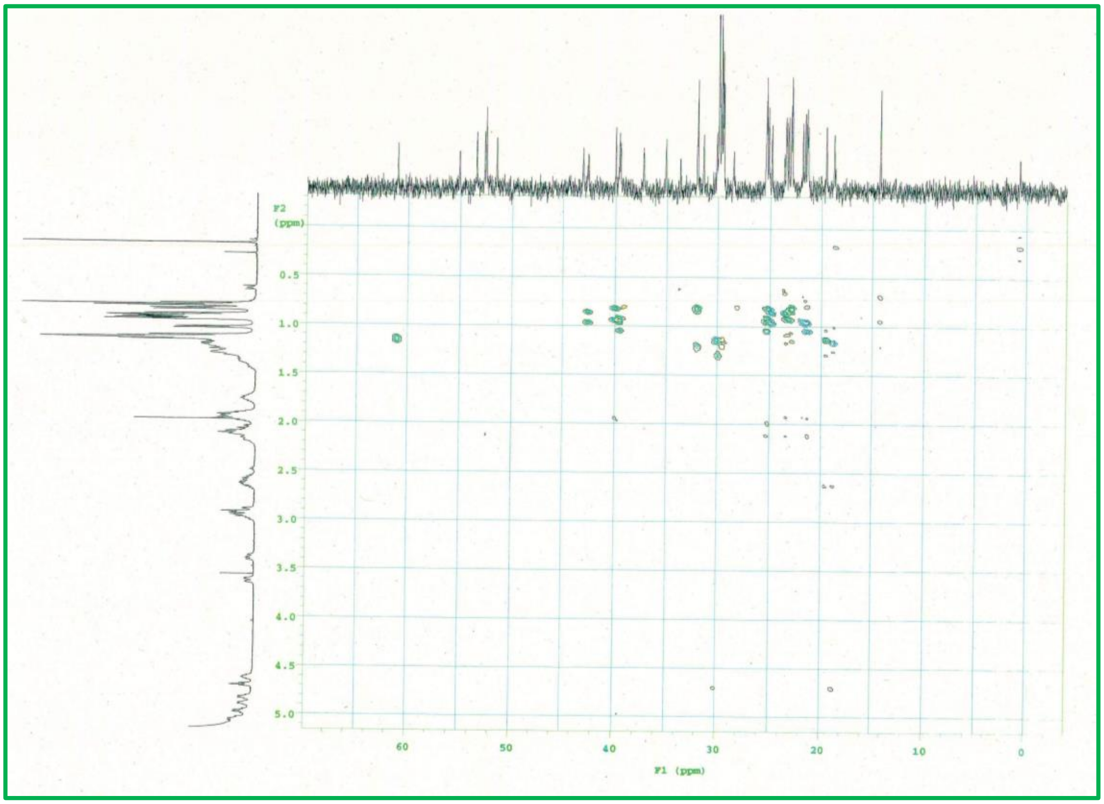


**Figure S7-e**. HMBC spectrum expansions of compound **1** (pyridine-$d_5$)

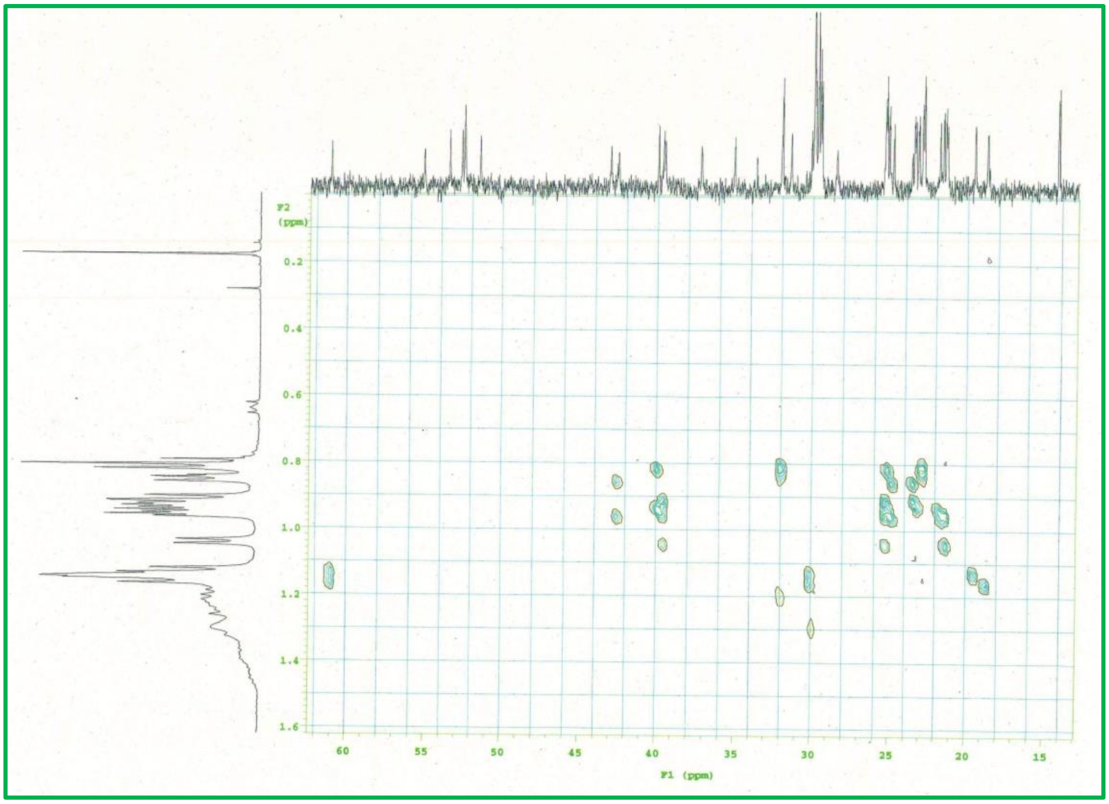


**Figure S7-f**. HMBC spectrum expansions of compound **1** (pyridine-$d_5$)

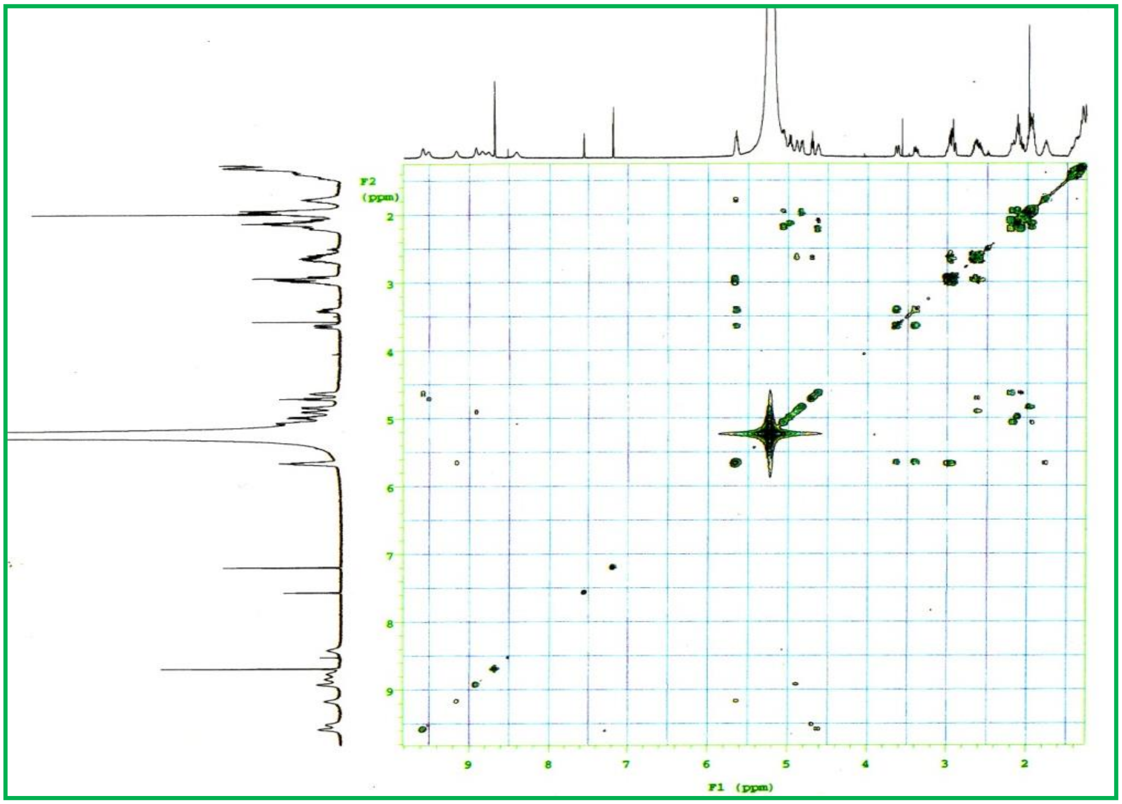


**Figure S8**. $^1H$-$^1H$ COSY correlations of compound **1** (pyridine-$d_5$)

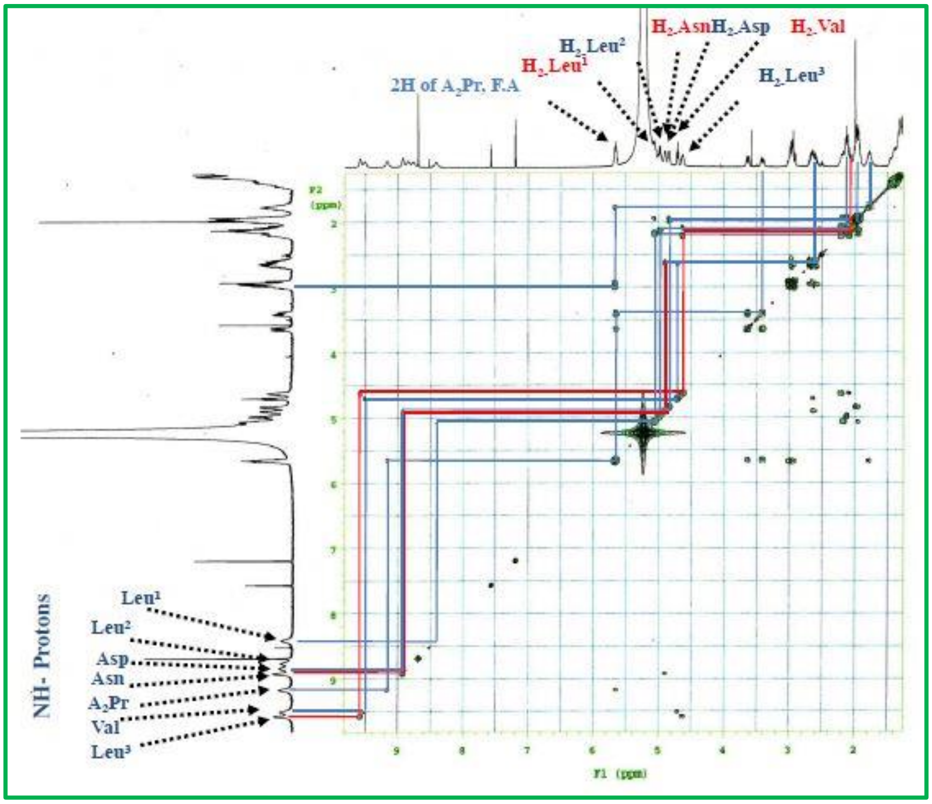


**Figure S8-a**. $^1H$-$^1H$ COSY spectrum of compound **1** with identified correlations

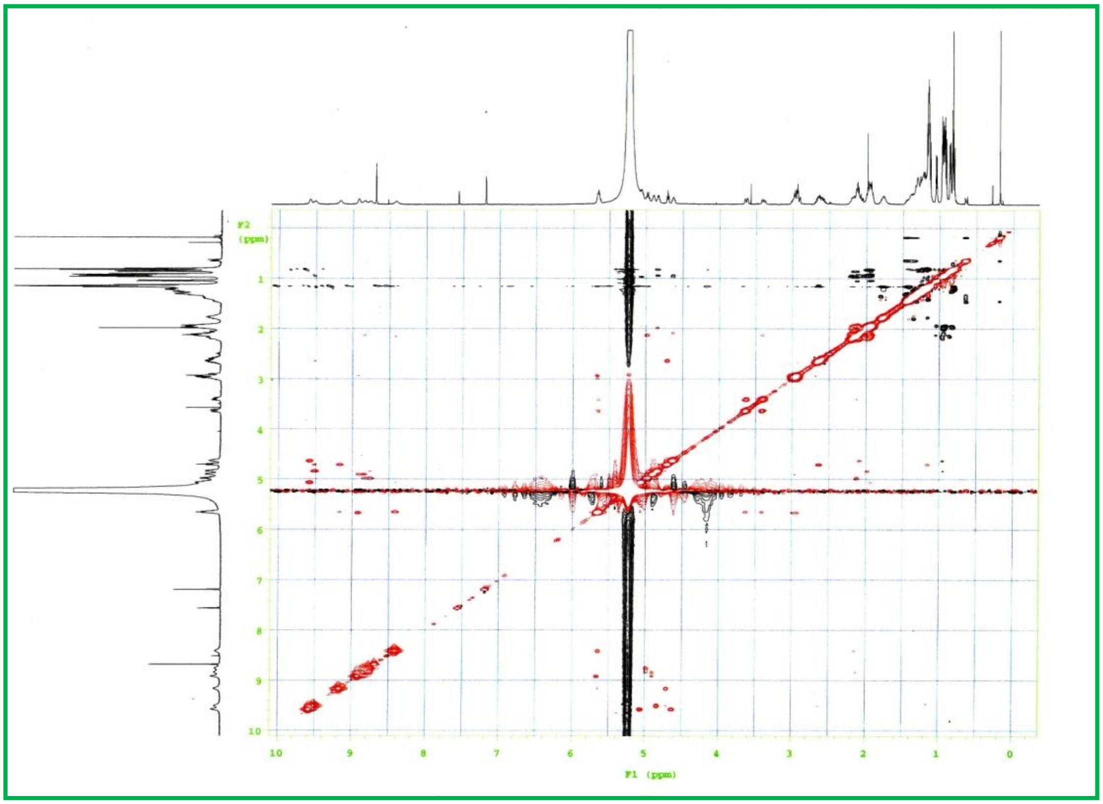


**Figure S9**. NOESY correlations of compound **1** (pyridine-$d_5$)

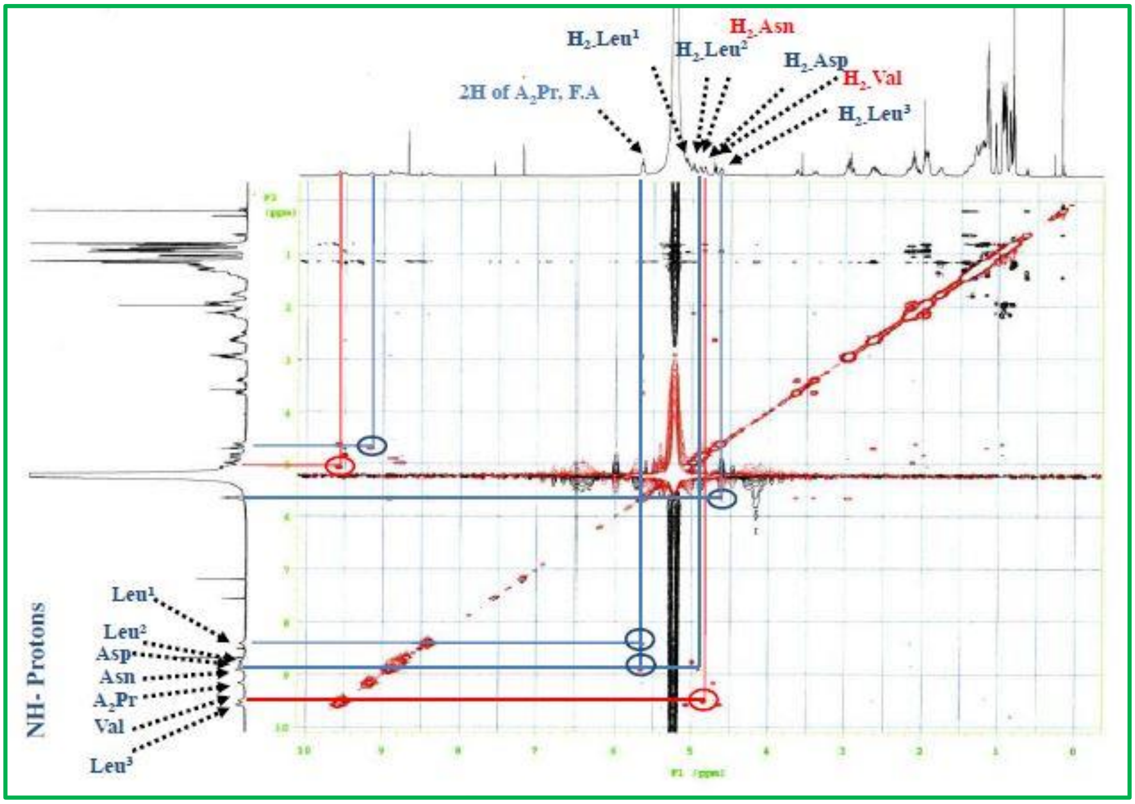


**Figure S9-a**. NOESY spectrum of compound **1** with identified correlations
